# Accuracy of Dynamic Navigation for Non-Surgical Endodontic Treatment: A Systematic Review

**DOI:** 10.3390/jcm11123441

**Published:** 2022-06-15

**Authors:** Egle Marija Jonaityte, Goda Bilvinaite, Saulius Drukteinis, Andres Torres

**Affiliations:** 1 Institute of Dentistry, Faculty of Medicine, Vilnius University, Zalgirio 115, 08217 Vilnius, Lithuania; egle.jonaityte@mf.stud.vu.lt (E.M.J.); goda.bilvinaite@mf.stud.vu.lt (G.B.); 2 OMFS-IMPATH Research Group, Department of Imaging and Pathology, Faculty of Medicine, University of Leuven, 3000 Leuven, Belgium; andres.torres@uzleuven.be; 3 Department of Oral Health Sciences, Endodontology, University Hospitals Leuven, Katholieke Universiteit Leuven, 3000 Leuven, Belgium

**Keywords:** endodontics, dynamic navigation, guided endodontics, real-time tracking

## Abstract

In recent years, the application of Guided Endodontics has gained interest for non-surgical endodontic treatment and retreatment. The newest research focuses on the accuracy of Dynamic Navigation (DN). This article systematically reviewed existing data on the accuracy of non-surgical endodontic treatment procedures that were completed using DN. Following the PRISMA criteria, an electronic database search was conducted in PubMed, Web of Science, Scopus, and Cochrane Library. Studies comparing the accuracy of non-surgical endodontic treatment using DN and the conventional freehand technique were eligible. The literature search resulted in 176 preliminary records. After the selection process six studies were included. The risk of bias was evaluated using the modified Cochrane Collaboration Risk of Bias 2.0 tool. Five studies examined the aid of DN for planning and executing endodontic access cavities, and one for fiber post removal. In two studies, endodontic access cavities were performed in teeth with pulp canal obliteration. The main outcomes that were measured in the included studies were preparation time, global coronal entry point and apical endpoint deviations, angular deviation, tooth substance loss, qualitative precision, number of unsuccessful attempts or procedural mishaps. The risk of bias was rated from low to raising some concerns. Overall, DN showed increased accuracy compared to the freehanded technique and could be especially helpful in treating highly difficult endodontic cases. Clinical studies are needed to confirm the published in vitro data.

## 1. Introduction

Traditionally, endodontic access cavity is prepared freehanded, according to the operator’s clinical experience and knowledge of tooth anatomy. The anatomical laws of the pulp chamber which were formulated by Krasner and Rankow are used to aid in locating the canal [1]. Moreover, a dental operating microscope can be used during this treatment step to reduce the possibility of iatrogenic mishaps [2]. However, some clinical conditions, such as canal obliteration can prolong the location of a canal up to 60 min even using a dental operating microscope [3]. Further, technical failures, including missed canals, crown or root perforations, canal transportation, or weakened tooth structure, can reduce treatment success or lead to tooth extraction [4,5]. Furthermore, due to some systemic conditions, e.g., patients taking bisphosphonates, tooth extraction is contraindicated, thus making locating even severely obliterated tooth canals essential in the case of apical periodontitis [6]. Therefore, to facilitate the management of difficult and complicated endodontic cases, the concept of Guided Endodontics was introduced [7]. This method allowed static navigation of the bur using a 3D printed template while preparing the endodontic access cavity. However, the concept has some drawbacks: increased planning time, the possible inaccuracies of pre-operative cone-beam computed tomographic (CBCT) or intra-oral scanning, difficult application in premolar and molar regions due to limited vertical space, and the requirement of straight-line access to the root canal [8]. These drawbacks limit the use of static guides to anterior teeth. 

In 2000, dynamic navigation (DN) was implemented to increase accuracy in dental implant placement by providing the operator with a real-time navigation tool [9]. DN uses preoperative CBCT data for pre-treatment virtual planning and real-time guidance of bur positioning during the procedure. Recently, DN gained interest in the field of Guided Endodontics as it has some advantages over static guides: it can be used in posterior regions, it allows a change in the drilling path due to real-time tracking, and the patient can be treated in the same appointment [8,10]. 

The aim of this study is to systematically review the available literature on the accuracy of non-surgical endodontic treatment procedures that are completed freehanded and using DN.

## 2. Materials and Methods

### 2.1. Study Design

The present systematic review was conducted in accordance with the PRISMA (Preferred Reporting Items for Systemic Reviews and Meta-Analyses) guidelines. The detailed PICO principles were defined as follows:

- Population—human teeth or three-dimensional (3D) printed teeth;
- Intervention—non-surgical endodontic treatment using the dynamic navigation system;
- Comparison—non-surgical endodontic treatment using the conventional freehand technique;
- Outcome—accuracy and efficiency of non-surgical endodontic treatment.

The protocol was registered in PROSPERO (International Prospective Register of Systematic Reviews; registration number of CRD42021287170).

### 2.2. Search Strategy

The relevant studies were searched in the following databases: PubMed, Web of Science, Scopus, and Cochrane Library, by two independent reviewers (E.M.J. and G.B.). The search covered all the literature that was published from the inception of each database to September 2021, with no language or regional restrictions. The search strategy used in PubMed was as follows: “Surgical navigation systems”[Mesh] OR “Dynamic navigation” OR “Guided endodontic” OR “Computer-assisted treatment” OR “Computer-aided navigation” OR “Image-guided treatment” OR “Navigation system” OR “Real-time tracking” OR “Dynamic guide” AND “Endodontics”[Mesh] OR “Root canal therapy”[Mesh] OR “Dental pulp calcification”[Mesh] OR “Dental pulp”[Mesh] OR “Dental pulp cavity”[Mesh] OR “Access cavit*” OR “Pulp canal calcification” OR “Root canal treatment” OR “Endodontic*” OR “Minimally invasive dentistry” OR “Obliterat*” OR “Conservative endodontic access” OR “minimally invasive access”. The same terms were used in adapted versions of the search strategy for each database. An additional manual search was performed to identify the potentially eligible studies that were not indexed in the databases mentioned above. 

### 2.3. Study Selection 

The titles, abstracts and full texts of the identified studies were independently screened for eligibility by two reviewers (E.M.J. and G.B.). Literature reviews and clinical cases were excluded at the initial stage of screening. The inclusion criteria involved the following:

- Randomized experimental trials (RETs) or clinical trials (RCTs);
- Non-surgical endodontic treatment using a dynamic navigation system;
- Outcomes compared to conventional freehand technique;
- Articles available in full text.

The exclusion criteria were as follows: case reports, reviews, non-English language articles, studies using CBCT as mean of navigation technique, performing surgical endodontic treatment or having no control group. 

The inter-reviewer agreement on the study selection was determined by the value of Cohen’s kappa. Any disagreement on the study selection was resolved by discussion until a consensus was reached. The third reviewer (S.D.) was involved when necessary.

### 2.4. Data Extraction

The data extraction from each eligible study was accomplished by two reviewers (E.M.J. and G.B.) separately. No differences between the collected information consisting of references (authors, year of publication, country), study design, sample size, type of teeth, measured parameters and results were observed at the end of data extraction. 

In cases of multiple experimental groups, the data conforming to PICO were collected. When the data were missing or unclear, the corresponding authors of the relevant studies were contacted. 

### 2.5. Quality Assessment

The quality of the selected studies was assessed by two independent reviewers (E.M.J. and G.B.) using the modified Cochrane Risk of Bias 2.0 tool (version 2, Cochrane Collaboration, London, UK) for randomized trials (RoB 2). All the domains (randomization process, deviations from the intended interventions, missing outcome data, measurement of the outcome and selection of the reported result) were classified as low, unclear or high risk of bias. Studies with at least one domain of a high risk of bias were overall rated as a high risk of bias. The unclear risk of bias was attributed to studies with no high- risk domains and at least one domain of unclear risk. 

The lack of agreement between the two reviewers was resolved by discussion with the third reviewer (S.D.).

## 3. Results

### 3.1. Study Selection

**Figure 1:**
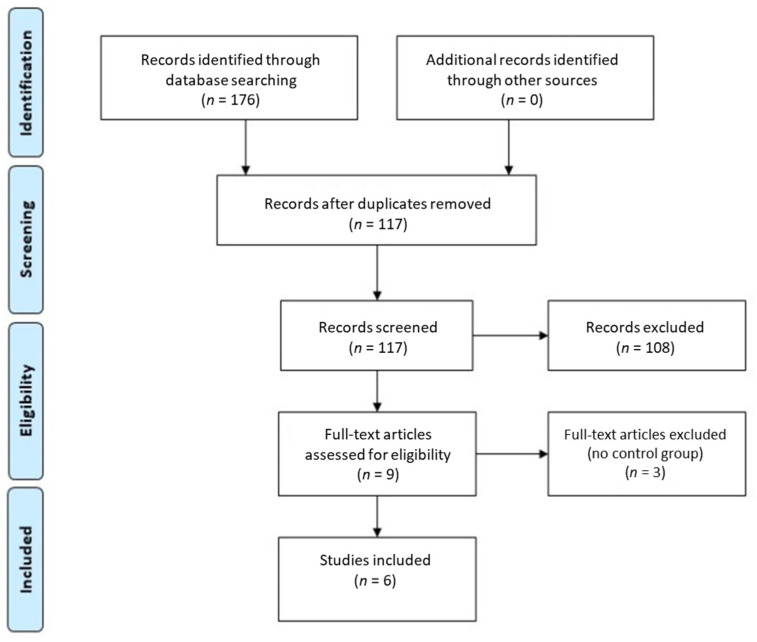
The review search and selection flowchart.

Our search identified an initial number of 176 articles. The selection strategy is shown in the PRISMA flow chart (Preferred Reporting Items for Systematic Reviews and Meta-Analyses) (Figure 1) [11]. After the elimination of duplicates, 117 articles were screened by the reviewers. After filtering through titles and reading the abstracts, nine articles were selected for full-text reading, and six articles were considered to be eligible for inclusion in this systematic review. Cohen’s κ-value for the inter-rater agreement was 0.92.

### 3.2. Study Characteristics

The main characteristics of the articles that were included in this review are summarized in Table 1. All the included articles were in vitro studies that were published in the years 2020 and 2021. Three studies used freshly extracted human teeth [12,13,14] and three used resin teeth [15,16,17]; the former studies used single-rooted teeth. Gambarini et al. [15] used resin upper first molars, whereas Connert et al. [17] and Jain et al. [16] used single-rooted printed teeth. The teeth in their correct anatomical position were either embedded in artificial jaw models [14,15,16,17] or in cadaver maxillae or mandibles [12,13]. Dianat et al. [13] selected teeth with pulp canal obliteration and Jain et al. [16] 3D printed teeth with stimulated canal obliteration. 

All the studies compared DN to conventional freehand preparation (FH) techniques, except Zubizarreta et al. [14], who also included a guided technique group. Five studies [13,14,15,16,17] examined the aid of DN for planning and executing endodontic access cavities and one for fiber post removal [12]. Two studies also compared the influence of the operator’s experience on the results [13,17].

The main outcomes measured were the accuracy and efficiency of the DN compared with the FH technique. Five studies compared the preparation time between DN and FH groups [12,13,15,16,17]. Various measures were used to determine the preparation accuracy, including coronal entry point and apical endpoint deviations [11,12,13,14], angular deviation [12,13,14,15], tooth substance loss [16,17], qualitative precision [16], the number of unsuccessful attempts or procedural mishaps [12,13,14,16,17].

### 3.3. Quality Assessment

**Figure 2:**
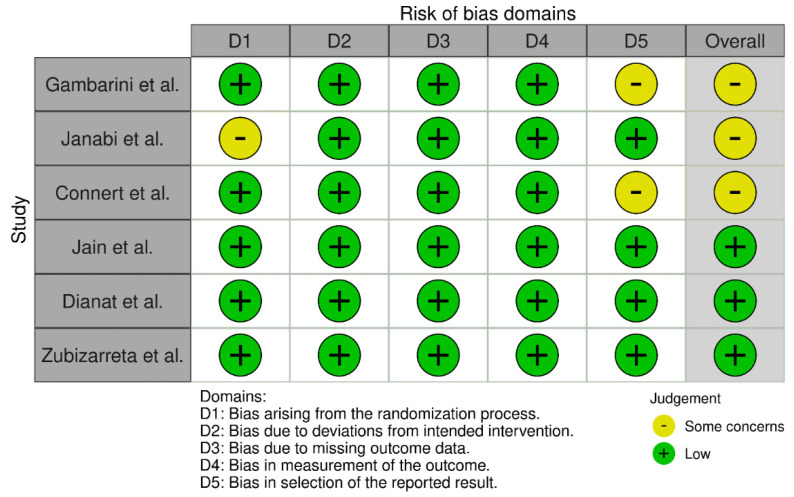
Risk of bias assessment using the modified RoB 2.0 tool. Gambarini et al. [15]; Janabi et al. [12]; Connert et al. [17]; Jain et al. [16]; Dianat et al. [13]; Zubi-zarreta et al., 2020 (Spain) [14].

Overall, the risk of bias was rated as low in three included studies [13,14,16] and as raising some concerns in the remaining three studies [12,15,17]. In addition, some concerns emerged from the randomization process [12] and the selection of the reported results [15,17]. Detailed results regarding the risk of bias of the included studies are presented in Figure 2.

## 4. Discussion

The present systematic review aimed to analyze the aid of DN to increase accuracy in endodontic procedures. It is now accepted that the loss of structural integrity that is associated with access cavity preparation and dentin removal, particularly in the peri-cervical region, are major causes of fracture in endodontically treated teeth [18]. Therefore, accurate access cavity preparation can reduce substance loss on endodontic treated tooth [12,16,17]. All the studies in this review reported increased accuracy and less volumetric loss of tooth structure when using DN. Furthermore, DN led to fewer iatrogenic errors. Among the studies, 119 teeth were treated using DN, in which two incidents of perforations and one case of gouging were reported. The most common procedural mishaps and errors were artifacts in the CBCT scan from restorations containing metal, planning errors, incorrect calibration, faulty transfer of the anatomic landmarks during registration, misfit of tracking components, inadequate systems check during the treatment and practitioner hand tremor [9,19]. Thus, it is essential to ensure accuracy at each step to avoid the accumulation of errors. Further, there is a long learning curve for the practitioner when working with the DN because the technique requires a certain level of technical skill, hand-eye coordination and manual dexterity [9,19,20]. Torres et al. [20] observed accuracy result differences between operators during training, however, there were no statistically significant differences when the post-training treatment was carried out. They concluded that training is essential to achieve predictable results. Only two studies, included in this review, reported using 20 teeth to train the operator before the experiment [12,13]. 

The time required to perform an endodontic treatment is essential for the patient and dental practitioner. Preparation time was recorded in five of the included studies [12,13,15,16,17]. Statistically significant differences in time between the DN and FH groups were found in the preparation of access cavities in teeth with root canal obliteration and fiber post removal [12,13,16]. Time differences between the studies can be explained by different measuring start and endpoints, simulated clinical situations, and research method differences. For example, Gambarini et al. [15] and Janabi et al. [12] did not specify start and end measurement points. In comparison, Jain et al. [16] used different endpoints of preparation time measurement for the FH and DN groups. The endpoint in the FH group was set as the successful canal negotiation or when the access depth was suspected to reach the estimated measurement to the canal space; the endpoint in the DN group was selected when the bur reached the end of the planned drill path. Root canal obliteration can be caused by dental trauma, carious lesions, orthodontic treatment, regenerative endodontic procedures and individual aging, and it is becoming more frequent [21,22]. Fiber posts have also been increasingly used to restore endodontically treated teeth because of high survival rates and improved esthetics, compared to metal posts [23,24]. According to the American Association of Endodontists (AAE), root canal obliteration and fiber post removal are considered to be high difficulty endodontic cases which should be considered for referral [25]. Dianat et al. [13] found that using DN for locating obliterated canals allowed to avoid tooth perforation. Consequently, DN could be a superior choice when dealing with clinically challenging cases. 

Two studies compared clinicians of different experience levels [13,17]. Connert et al. [17] found that less experienced operators removed significantly more tooth structure using the FH technique than more experienced clinicians. There were no statistically significant differences between the operators when using DN. Dianat et al. reported a statistically significant difference between a board-certified endodontist and a third-year endodontic resident for the time that was required to locate the canal using the FH technique [13]. Again, there was no statistically significant difference in the DN group. Torres et al. [20] compared three operators with varying experience levels for access cavity preparation in teeth with severe root canal obliteration using DN. They found that 93% of canals were located irrespective of the operator’s experience in endodontics, after appropriate training sessions with the device. These results suggest that DN can be beneficial for novice practitioners to combat high difficulty endodontic cases. Moreover, some studies evaluated the impact of DN on training dental students in dental implant placement. The results show that DN can be a valuable tool to improve the training of novice operators [26,27]. 

Half of the included studies used extracted human teeth [12,13,14], while the other half used 3D printed tooth replicas [15,16,17] for the experiments. Natural extracted human teeth contain anatomical landmarks, such as a pulp chamber floor map and tertiary dentin color, which are important for freehanded endodontic access preparation and obliterated root canal location. In contrast, 3D printed resin teeth do not possess such qualities [1]. Therefore, a freehanded search for obliterated root canals in 3D printed teeth can be misleading. Moreover, the operator can become familiar with tooth anatomy and canal location. To overcome this drawback of 3D printed teeth, Jain et al. [16] recommend at least a one-week interval between treatment sessions. 

Since DN was first introduced for dental implant placement, many studies have evaluated the accuracy and efficiency of DN in dental implantology, both in vitro and in clinical investigations. A recent systematic review and meta-analysis [28], which included in vitro and clinical studies, reported that clinical studies demonstrate slightly higher deviations than in vitro studies. The mean overall angular deviations were 2.01° (95% CI: 1.95 to 2.07) in in vitro studies and 3.68° (95% CI: 3.61 to 3.74) in clinical studies. Further, more than 1 mm deviations were observed in some clinical studies. These deviations can be of great importance in endodontics. However, currently there are just a few case reports of applying DN for access cavity preparation and endodontic microsurgery [29,30,31,32]. Therefore, clinical trials are necessary to confirm the published in vitro data of DN accuracy in endodontics.

Only one included study compared DN with static guidance [14]. Static guides are 3D printed templates which are manufactured using preoperative CBCT and intraoral scanning data [7]. Results show that DN was more accurate than a static guide for endodontic access cavity preparation in an absolute value. However, the results showed no statistically significant differences.

The strength of the present systematic review is robust inclusion criteria, which were used to focus on the topic and decrease the possibility of bias arising from study selection. Another advantage is the overall low risk of bias of the included studies. Therefore, the limitations include the possibility of missing related articles, although this was decreased by searching four databases. Other potential limitations are the small number of included studies, the range of the study designs and the outcome measures which impede comparison. Although a meta-analysis was not attempted due to these limitations, this systematic review can provide some directions for the near future to standardize outcome measures.

## 5. Conclusions

Within the limitations of this systematic review, it can be concluded that the dynamic navigation system demonstrated increased accuracy, compared to the freehanded technique, and can be helpful in managing complicated endodontic cases after proper training with the device. However, well-designed clinical trials are necessary to confirm published in vitro data in the future.

## Figures and Tables

**Table 1:** Studies characteristics and results.

Study	Sample Size	DN System	Specimens	Outcome Measure	DN Technique Results ± SD (95% CI)	FH Technique Results ± SD (95% CI)
Gambarini et al., 2020 (Italy) [15]	20 (*n* = 10)	Navident (ClaroNav)	Artificial, made of resin upper right first molars.	Preparation time. Maximum distance between planned and prepared access cavity at the orifice level. Access cavity angular deviation. Ability to locate a canal.	11.5 ± 2.4 s 0.34 ± 0.19 mm * DN 4.8° ± 1.8° * All canals were located.	12.2 ± 3.2 s 0.88 ± 0.41 mm * 19.2° ± 8.9° * All canals were located.
Janabi et al., 2021 (USA) [12]	26 (*n* = 13)	X-guide system (X-Nav Technologies)	Extracted human maxillary single-rooted teeth (incisors and canines). Teeth were endodontically treated and restored with fiber post.	Preparation time. Drilling trajectory. global coronal deviation. Drilling trajectory global apical deviation. Access cavity angular deviation. The volume of tooth structure before and after preparation. Procedural mishaps.	241.8 ± 25.8 s * 0.91 ± 0.65 mm * 1.17 ± 0.64 mm * 1.75° ± 0.63° * Before 542.50 ± 81.97 mm^3^; After 487.87 ± 74.70 mm^3^ * No perforations.	498 ± 279 s * 1.13 ± 0.83 mm * 1.68 ± 0.85 mm * 4.49° ± 2.10° * Before 571.34 ± 133.12 mm^3;^ After 533.16 ± 133.12 mm^3^ * No perforations.
Connert et al., 2021 (Switzerland) [17]	72 (*n* = 18)	DENACAM system (Mininavident AG)	3D printed using resin maxillary single-rooted teeth (incisors and canines).	Preparation time. Tooth substance volume loss. Procedural mishaps.	195 (135–254) s 10.5 (7.6–13.3) mm^3^ * One perforated canal.	193 (164–222) s 29.7 (24.2–35.2) mm^3^ * One perforated canal.
Jain et al., 2020 (USA) [16]	40 (*n* = 20)	Navident (ClaroNav)	3D printed single-rooted teeth with simulated pulp canal obliteration (maxillary and mandibular central incisors).	Preparation time. Tooth substance volume loss. Qualitative precision: optimal, suboptimal or unacceptable.	136.1 (101.4–170.8) s * 27.2 (22.0–32.5) mm^3^ * 75% optimal; 15% suboptimal; 10% unacceptable (one perforation)	424.8 (289.4–560.2) s * 40.7 (29.1–52.2) mm^3^ * 45% optimal; 40% suboptimal; 15% unacceptable (two perforations)
Dianat et al., 2020 (USA) [13]	60 (*n* = 15)	X-Guide system (X-Nav Technologies)	Extracted human single-rooted teeth with pulp canal obliteration (maxillary and mandibular incisors, canines and premolars).	Preparation time. Access cavity linear deviation (in the BL and MD directions). Reduced dentin thickness (at the CEJ level and at the end of the drilling point (EDP)). Access cavity angular deviation. Successfully located canals. Procedural mishaps.	227 ± 97 s * BL 0.19 ± 0.21 mm *MD 0.12 ± 0.14 mm CEJ 1.06 ± 0.18 mm *; EDP 1.18 ± 0.17 mm * 2.39° ± 0.85° * 96.6% (29/30) One gouging *	405 ± 246 s * BL 0.81 ± 0.74 mm *; MD 0.31 ± 0.35 mm CEJ 1.55 ± 0.55 mm *; EDP 1.47 ± 0.49 mm * 7.25° ± 4.2° * 83.3% (25/30) Five perforations, three gouging *
Zubizarreta et al., 2020 (Spain) [14]	30 (*n* = 10)	Navident (ClaroNav)	Extracted human single-rooted teeth (lower central incisors).	Access cavity angular deviation. Access cavity linear deviation (measured at the coronal entry point (CEP) and the end of the drilling point (EDP).	5.58° ± 3.23° * CEP 3.14 ± 0.86 mm *; EDP 2.48 ± 0.94 mm *	14.95° ± 11.15° * CEP 4.03 ± 1.93 mm *; EDP 2.43 ± 1.23 mm *

* Significant pair-wise comparison between DN and FH Techniques.

## Data Availability

Not applicable.

## References

[1] Krasner P., Rankow H.J. (2004). Anatomy of the pulp-chamber floor. J. Endod..

[2] Iandolo A., Iandolo G., Malvano M., Pantaleo G., Simeone M. (2016). Modern technologies in Endodontics. G. Ital. Endod..

[3] Kiefner P., Connert T., ElAyouti A., Weiger R. (2017). Treatment of calcified root canals in elderly people: A clinical study about the accessibility, the time needed and the outcome with a three-year follow-up. Gerodontology.

[4] Karabucak B., Bunes A., Chehoud C., Kohli M.R., Setzer F. (2016). Prevalence of Apical Periodontitis in Endodontically Treated Premolars and Molars with Untreated Canal: A Cone-beam Computed Tomography Study. J. Endod..

[5] Touré B., Faye B., Kane A.W., Lo C.M., Niang B., Boucher Y. (2011). Analysis of Reasons for Extraction of Endodontically Treated Teeth: A Prospective Study. J. Endod..

[6] Song M. (2019). Dental care for patients taking antiresorptive drugs: A literature review. Restor. Dent. Endod..

[7] Zehnder M.S., Connert T., Weiger R., Krastl G., Kühl S. (2016). Guided endodontics: Accuracy of a novel method for guided access cavity preparation and root canal location. Int. Endod. J..

[8] Connert T., Weiger R., Krastl G. (2022). Present status and future directions—Guided endodontics. Int. Endod. J..

[9] Panchal N., Mahmood L., Retana A., Emery R. (2019). Dynamic Navigation for Dental Implant Surgery. Oral Maxillofac. Surg. Clin..

[10] Chong B.S., Dhesi M., Makdissi J. (2019). Computer-aided dynamic navigation: A novel method for guided endodontics. Quintessence Int..

[11] Page M.J., McKenzie J.E., Bossuyt P.M., Boutron I., Hoffmann T.C., Mulrow C.D., Shamseer L., Tetzlaff J.M., Akl E.A., Brennan S.E. (2021). The PRISMA 2020 statement: An updated guideline for reporting systematic reviews. Syst. Rev..

[12] Janabi A., Tordik P.A., Griffin I.L., Mostoufi B., Price J.B., Chand P., Martinho F.C. (2021). Accuracy and Efficiency of 3-dimensional Dynamic Navigation System for Removal of Fiber Post from Root Canal-Treated Teeth. J. Endod..

[13] Dianat O., Nosrat A., Tordik P.A., Aldahmash S.A., Romberg E., Price J.B., Mostoufi B. (2020). Accuracy and Efficiency of a Dynamic Navigation System for Locating Calcified Canals. J. Endod..

[14] Zubizarreta-Macho Á., Muñoz A.P., Deglow E.R., Agustín-Panadero R., Álvarez J.M. (2020). Accuracy of Computer-Aided Dynamic Navigation Compared to Computer-Aided Static Procedure for Endodontic Access Cavities: An In Vitro Study. J. Clin. Med..

[15] Gambarini G., Galli M., Morese A., Stefanelli L.V., Abduljabbar F., Giovarruscio M., Di Nardo D., Seracchiani M., Testarelli L. (2020). Precision of Dynamic Navigation to Perform Endodontic Ultraconservative Access Cavities: A Preliminary In Vitro Analysis. J. Endod..

[16] Jain S.D., Saunders M.W., Carrico C.K., Jadhav A., Deeb J.G., Myers G.L. (2020). Dynamically Navigated versus Freehand Access Cavity Preparation: A Comparative Study on Substance Loss Using Simulated Calcified Canals. J. Endod..

[17] Connert T., Leontiev W., Dagassan-Berndt D., Kühl S., ElAyouti A., Krug R., Krastl G., Weiger R. (2021). Real-Time Guided Endodontics with a Miniaturized Dynamic Navigation System Versus Conventional Freehand Endodontic Access Cavity Preparation: Substance Loss and Procedure Time. J. Endod..

[18] Schwartz R.S., Robbins J.W. (2004). Post placement and restoration of endodontically treated teeth: A literature review. J. Endod..

[19] Jain S.D., Carrico C.K., Bermanis I., Rehil S. (2020). Intraosseous Anesthesia Using Dynamic Navigation Technology. J. Endod..

[20] Torres A., Boelen G.J., Lambrechts P., Pedano M.S., Jacobs R. (2021). Dynamic navigation: A laboratory study on the accuracy and potential use of guided root canal treatment. Int. Endod. J..

[21] de Toubes K.M.P.S., de Oliveira P.A.D., Machado S.N., Pelosi V., Nunes E., Silveira F.F. (2017). Clinical approach to pulp canal obliteration: A case series. Iran Endod. J..

[22] Carvalho T.S., Lussi A. (2017). Age-related morphological, histological and functional changes in teeth. J. Oral Rehabil..

[23] Dikbas I., Tanalp J. (2013). An Overview of Clinical Studies on Fiber Post Systems. Sci. World J..

[24] Garcia P.P., Wambier L.M., de Geus J.L., da Cunha L.F., Correr G.M., Gonzaga C.C. (2019). Do anterior and posterior teeth treated with post-and-core restorations have similar failure rates? A systematic review and meta-analysis. J. Prosthet. Dent..

[25] American Association of Endodontists AAE Endodontic Case Difficulty Assessment Form and Guidelines. https://www.aae.org/specialty/wp-content/uploads/sites/2/2019/02/19AAE_CaseDifficultyAssessmentForm.pdf.

[26] Zhan Y., Wang M., Cheng X., Li Y., Shi X., Liu F. (2021). Evaluation of a dynamic navigation system for training students in dental implant placement. J. Dent. Educ..

[27] Golob Deeb J., Bencharit S., Carrico C.K., Lukic M., Hawkins D., Rener-Sitar K., Deeb G.R. (2019). Exploring training dental implant placement using computer-guided implant navigation system for predoctoral students: A pilot study. Eur. J. Dent. Educ..

[28] Jorba-García A., González-Barnadas A., Camps-Font O., Figueiredo R., Valmaseda-Castellón E. (2021). Accuracy assessment of dynamic computer–aided implant placement: A systematic review and meta-analysis. Clin. Oral. Investig..

[29] Gambarini G., Galli M., Stefanelli L.V., Di Nardo D., Morese A., Seracchiani M., De Angelis F., Di Carlo S., Testarelli L. (2019). Endodontic Microsurgery Using Dynamic Navigation System: A Case Report. J. Endod..

[30] Dianat O., Gupta S., Price J.B., Mostoufi B. (2021). Guided Endodontic Access in a Maxillary Molar Using a Dynamic Navigation System. J. Endod..

[31] Bardales-Alcocer J., Ramírez-Salomón M., Vega-Lizama E., López-Villanueva M., Alvarado-Cárdenas G., Serota K.S., Ramírez-Wong J. (2021). Endodontic Retreatment Using Dynamic Navigation: A Case Report. J. Endod..

[32] Lu Y.J., Chiu L.H., Tsai L.Y., Fang C.Y. (2022). Dynamic navigation optimizes endodontic microsurgery in an anatomically challenging area. J. Dent. Sci..

